# An exploration of the use of simple statistics to measure consensus and stability in Delphi studies

**DOI:** 10.1186/1471-2288-7-52

**Published:** 2007-11-29

**Authors:** Elizabeth A Holey, Jennifer L Feeley, John Dixon, Vicki J Whittaker

**Affiliations:** 1 School of Health and Social Care, University of Teesside, Middlesbrough, TS1 3BA, UK; 2 Department of Physiotherapy, Kings Mill Hospital, Mansfield Road, Sutton-In-Ashfield, Nottinghamshire, NG17 4JT, UK

## Abstract

**Background:**

The criteria for stopping Delphi studies are often subjective. This study aimed to examine whether consensus and stability in the Delphi process can be ascertained by descriptive evaluation of trends in participants' views.

**Methods:**

A three round email-based Delphi required participants (n = 12) to verify their level of agreement with 8 statements, write comments on each if they considered it necessary and rank the statements for importance. Each statement was analysed quantitatively by the percentage of agreement ratings, importance rankings and the amount of comments made for each statement, and qualitatively using thematic analysis. Importance rankings between rounds were compared by calculating Kappa values to observe trends in how the process impacts on subject's views.

**Results:**

Evolution of consensus was shown by increase in agreement percentages, convergence of range with standard deviations of importance ratings, and a decrease in the number of comments made. Stability was demonstrated by a trend of increasing Kappa values.

**Conclusion:**

Following the original use of Delphi in social sciences, Delphi is suggested to be an effective way to gain and measure group consensus in healthcare. However, the proposed analytical process should be followed to ensure maximum validity of results in Delphi methodology for improved evidence of consensual decision-making.

## Background

The Delphi technique is suggested to be an effective way to gain and measure group consensus in healthcare [1]. Delphi was developed by Dalkey and colleagues at the RAND Corporation in the 1950's, and is a structured process requiring experts to respond to non-leading, unambiguous statements with the aim of achieving consensus. Using a systematic fashion of repeating rounds, where each subsequent set of statements is built on the responses to the preceding ones, consensus is sought through the feedback of information and iteration [2], and the process is terminated when consensus is reached. Anonymity offered by Delphi can reduce the inhibition normally occurring in decision-making as individuals will be more open with their answers.

Although originally used as a methodological tool in social sciences, recently there has been a rise in the use of Delphi in healthcare research [3-9]. Delphi characteristics have been consistently described in the definitive texts [2,10,11], and in the paper by Caws [12], which considered consensus theories in healthcare. Importantly however, there is no general agreement in the literature that defines specific criteria to use to determine when consensus has been achieved, i.e. when to stop a Delphi study. Evidence on the evaluation of Delphi consensus is limited; researchers have not yet described how to determine when an exact level of the consensus is reached in Delphi.

Delphi studies have been used to develop and identify consensus by experts on a given topic. As interest has grown in the analysis of the data produced by this method, authors have attempted to clarify the relevant concepts.

It has been suggested that consensus is the same as agreement and that agreement can be determined by:

1) the aggregate of judgements (the pool of individual judgements) [2],

2) a move to a subjective level of central tendency [13],

3) or alternatively, by confirming stability, which is "the consistency of answers between successive rounds of the study [13]."

The first of these occurs within each Delphi round. The second and third occur between rounds. Researchers have been inconsistent in their use of these concepts. In addition some researchers support the use of pre-determined levels of consensus to reduce research bias [14] whilst others argue that applying numerical values to subjective responses gives an unconvincing analysis [15].

Scheibe *et al.*[16] suggest that stability should be used in Delphi studies to compare the views of participants, as they believed that reporting a percentage of expressed views does not reflect the nature of Delphi to look for resistance to natural centralisation of views. They also note complete stability will be difficult to attain as there will always be some "oscillatory movements." A subsequent study used stability rather than agreement as termination criteria for Delphi [13]. Dajani et al. [13] propose the use of Chi-squared (χ^2^) to test for stability. However, this cannot be considered to test stability in Delphi studies as it will determine "the independence of the rounds from responses found in them" [13] not the stability of responses between separate rounds. A study by Chaffin and Talley [17] using χ^2 ^to determine individual stability prior to group stability was developed from the work of Dajani et al., and therefore this should also be viewed with caution. Since these works few examples of χ^2 ^as a stability measure appear in Delphi literature. More recently Greatorex & Dexter used means and standard deviations (SD) for comparing movement between Delphi rounds as a measure of both stability and convergence [18].

In 1998 the NHS Health Technology Assessment group produced a detailed report on the requirements of effective consensus development methods [1] yet could not identify an appropriate statistical measure for reporting a move towards consensus, identified by central tendency in Delphi. They suggested reasoned feedback was advisable as well as central tendency measures but did not identify a statistical method which could do this. This lack of unambiguous criteria for defining consensus clearly shows that further research is required in this area.

### Aim of the Study

In order to reduce the subjectivity in stopping criteria used in Delphi studies, this study aimed to examine whether consensus and stability in the Delphi process can be ascertained by descriptive evaluation of trends in participants' views. We evaluated the evolution of consensus and stability by examining agreement percentages, importance rankings (based on simple descriptive statistics) and Kappa values. These were used to explore how quantitative results could inform Delphi users, firstly on the production of central tendency/consensus, and secondly on stability, thus reducing subjectivity in reporting Delphi results. The use of simple descriptive statistics, as used in previous work [18], makes this method very user-friendly.

## Methods

An email-based Delphi study was undertaken and comparative statistical testing applied. Ethical approval was obtained from University of Teesside and informed consent obtained from each participant. A convenience heterogeneous sample (n = 12) of volunteer nursing, occupational therapy and physiotherapy students. A three round email-based Delphi study was undertaken following a pilot, which checked whether the statements were clear, unambiguous and non-leading. Statements were taken directly from the paper by McCallin [19] which reviewed interdisciplinary practice, a topic in which all students were equivalent experts due to their experiences as students. The views and opinions reached were irrelevant to the studying question, which explored the change of the opinions towards consensus, rather than the nature of the consensus itself. In each round, participants were invited to respond by scaling each statement on degree of agreement and commenting on each statement as desired, (see example in Table 1), and finally ranking the statements in order of importance, (see example in Table 2).

**Table 1:** Example of lay-out as presented for statement 1, round 1

**1. Role definition is a significant factor underpinning successful teamwork.**	
Agreement	□ Strongly agree
	□ Agree
	□ No opinion
	□ Disagree
	□ Strongly disagree
Comments...	

**Table 2:** Example of lay-out as given for participants to rank importance of statements at the end of round 1

Rank	Role definition is a significant factor underpinning successful teamwork.
Rank	Collaboration relies on changing attitudes.
Rank	Collective understanding develops when health professionals have opportunity to get to know each other in a more personal sense.
Rank	The frequency of team meetings is the single most critical factor that fosters collaborative teamwork.

Between rounds views were analysed using Colazzi's 7-stage thematic analysis, modified from Holloway & Wheeler [20]. Quantitative analysis of the Delphi included calculations of:

1. Percentage response rates,

2. Percentages for each level of agreement for each statement to compensate for varying response rates,

3. Median, range and their associated group rankings using the importance ratings,

4. Mean (SD) and their associated group rankings using the importance ratings,

5. Weighted Kappa (K) values to compare chance-eliminated agreement between rounds.

For the following round, statements were rephrased if appropriate with the aim of moving towards consensus, based on the level of agreement and the majority theme highlighted. Successive rounds consisted of statements (some rephrased) percentage agreement levels, anonymous feedback from the previous round to show the range of views received, and the importance rankings using the median rank values. Participants were requested to read the feedback before responding again to statements.

Scaling methods of agreement and importance were adapted from Sim & Wright [21] and have been previously used and described in Delphi literature [2,22,23]. Agreement scales were particularly relevant as they gave an opportunity for participants to scale each statement independently. To make comparisons between rounds and for feedback, percentage agreements were calculated for each level of the scale to compensate for varying response rates.

Weighted Kappa (K) statistics were calculated for the within-subject level of agreement in their importance rankings between two rounds, not the level of agreement between participants. K values show a chance-corrected proportional agreement [24]. A weighted Kappa was appropriate as un-weighted does not take into account the magnitude of discrepancies between disagreements [25,26]. SPSS was unable to calculate the K-values because of the requirement for a weighted Kappa. Therefore Excel spreadsheets and handwritten crosstabs were used based on the descriptions by Armitage *et al*. [27] and interpretation by Anthony [28], Table 3 (see Fliess [29] for a full mathematical explanation and justification of the Kappa validity). K-values were used to compare agreement of importance rankings between rounds for each statement and Chaffin & Talley [17] stated an "individual stability test for Delphi studies provides more information than a group stability test," therefore justifying the use of Kappa.

**Table 3:** Adapted from Anthony (1999) showing the level of agreement represented by K-values.

**K-value**	**Agreement level**
0.0–0.2	Poor agreement
0.21–0.4	Fair agreement
0.41–0.6	Moderate agreement
0.61–0.8	Substantial agreement
0.81–1	Almost perfect agreement

## Results

The results section summarises the Delphi in terms of how consensus and stability evolved through rounds 1 to 3 by looking at the: -

• Agreement percentages,

• Importance rankings,

• Statement evolution,

• Theme production,

• Kappa values.

Agreement values were affected in the second round by a reduced response of 83% (10 of 12).

### Statement 1 (Table 4)

**Table 4:** Agreement and importance values for statement 1

		Round 1	Round 2	Round 3
**Agreement**	Strongly agree	42%	40%	50%
	Agree	42%	60%	50%
	No opinion	8%	0%	0%
	Disagree	8%	0%	0%
	Strongly disagree	0%	0%	0%
**Importance**	Median	3	2	2
	Range	3–7	1–4	1–3
	Rank	2	2	2
	Mean	3.33	2.20	2.00
	Standard deviation	1.78	1.03	0.60
	Rank	2	2	2

The main themes highlighted remained constant throughout the three rounds but decreased in duplication over the rounds. The range interval and SD decreased to low levels (difference of 2 and 0.60 respectively). By the end this statement had the lowest range and SD, suggesting greatest stability and consensus, also it had a low number of comments (3). When the median and mean were equal the range, 1–3, and SD, 0.60 were low. Importance ranking was always high (second place).

### Statement 2 (Table 5)

**Table 5:** Agreement and importance values for statement 2

		Round 1	Round 2	Round 3
**Agreement**	Strongly agree	27%	0%	25%
	Agree	55%	100%	67%
	No opinion	9%	0%	8%
	Disagree	9%	0%	0%
	Strongly disagree	0%	0%	0%
**Importance**	Median	4	4	4
	Range	1–8	2–7	3–8
	Rank	4	4 =	4
	Mean	4.58	4.10	4.33
	Standard deviation	2.50	1.45	1.78
	Rank	4	4	4

There was a variety of themes generated (6) in round 1, suggesting divergence, changing to 3 themes in round 2, then 2 in round 3, suggesting convergence.

For statement 2 there was an increase in agreement between round 1 and 2, then divergence rather than convergence occurred between rounds 2 and 3. Based on the SD there was less consensus for this statement in round 3.

### Statement 3 (Table 6)

**Table 6:** Agreement and importance values for statement 3

		Round 1	Round 2	Round 3
**Agreement**	Strongly agree	8%	0%	0%
	Agree	50%	70%	59%
	No opinion	0%	0%	8%
	Disagree	42%	20%	33%
	Strongly disagree	0%	10%	0%
**Importance**	Median	4.5	6	6
	Range	1–8	5–8	5–8
	Rank	5	6	6
	Mean	5.42	6.50	6.42
	Standard deviation	2.27	1.18	1.08
	Rank	7	8	8

New themes were generated in round three, also suggestive of instability. Interestingly, the two non-responders from round 2 disagreed with the third round statement affecting the percentage of agreement. This was 1 of only 2 statements showing disagreement in round 3 and showing an increase in disagreement between any rounds. Mean and median ranks both show this statement became less important to the participants, yet there was always a 2-interval difference between these ranks.

### Statement 4 (Table 7)

**Table 7:** Agreement and importance values for statement 4

		Round 1	Round 2	Round 3
**Agreement**	Strongly agree	8%	80%	58%
	Agree	42%	0%	42%
	No opinion	8%	0%	0%
	Disagree	42%	20%	0%
	Strongly disagree	0%	0%	0%
**Importance**	Median	5	3	3
	Range	2–8	2–6	1–4
	Rank	6	3	3
	Mean	4.83	3.70	3.00
	Standard deviation	2.04	1.64	0.95
	Rank	5	3	3

Four themes were highlighted in round one. Despite the change in agreement between rounds two and three only one comment was given in round three, the least number of comments for any statement throughout the Delphi. An increase in those strongly agreeing (from 8% to 80%) was observed for round 2, as shown in Table 7. The level of those strongly agreeing lowered in round 3 (to 58%). Mean and median values both showed increase in importance rankings. As in statement 1, when median and mean were equal, range and S.D were lowest.

### Statement 5 (Table 8)

**Table 8:** Agreement and importance values for statement 5

		Round 1	Round 2	Round 3
**Agreement**	Strongly agree	25%	10%	17%
	Agree	42%	60%	58%
	No opinion	25%	20%	17%
	Disagree	9%	10%	8%
	Strongly disagree	0%	0%	0%
**Importance**	Median	3.5	4	5.5
	Range	1–8	2–7	3–8
	Rank	3	4 =	5
	Mean	4.25	4.40	5.67
	Standard deviation	2.53	1.78	1.72
	Rank	3	5	5

New themes were generated in round 3 suggesting stability had not yet occurred.

For this statement agreement percentages and ranges did not vary appreciably still showing disagreement in round 3, suggesting stability. Although only changing by 0.06 between rounds 2 and 3, the SD suggests there is still convergence. The statement became less important, ranked 3 to 5.

### Statement 6 (Table 9)

**Table 9:** Agreement and importance values for statement 6

		Round 1	Round 2	Round 3
**Agreement**	Strongly agree	25%	10%	25%
	Agree	42%	60%	58%
	No opinion	0%	0%	17%
	Disagree	33%	20%	0%
	Strongly disagree	0%	10%	0%
**Importance**	Median	5.5	7	7
	Range	1–7	3–8	3–8
	Rank	7	8	7
	Mean	4.83	6.40	6.33
	Standard deviation	2.21	1.65	1.56
	Rank	6	7	7

Round 1 produced 5 themes. In round 3 all responses reflected different themes, previous themes and new themes suggesting the views of participants had not been exhausted. Agreement increased over the 3 rounds, inversely to the ranking of importance. Both the median and the mean define a move towards lesser importance with the progression of rounds. The range intervals of 6, 5 and 5, over the rounds suggest consensus was weak yet stability had occurred but the SD was still lowering suggesting continued convergence.

### Statement 7 (Table 10)

In round 1 there were 2 themes, which were reflected again in rounds 2 and 3, although the amount of participants responding decreased to 3 in both round 2 and round 3. This statement was consistently high on agreement and importance always ranked first. However, range interval of 4, and SD of 1.17, were not the lowest observed in round 3, suggesting the possibility of further convergence.

**Table 10:** Agreement and importance values for statement 7

		Round 1	Round 2	Round 3
**Agreement**	Strongly agree	50%	70%	67%
	Agree	42%	30%	33%
	No opinion	8%	0%	0%
	Disagree	0%	0%	0%
	Strongly disagree	0%	0%	0%
**Importance**	Median	2	1	1
	Range	1–7	1–6	1–5
	Rank	1	1	1
	Mean	2.67	1.90	1.50
	Standard deviation	1.97	1.73	1.17
	Rank	1	1	1

### Statement 8 (Table 11)

Only 4 comments were obtained in round 1 all reflecting agreement. Round 2 instigated only three comments, still reflecting agreement. This was the only statement for which the number of comments increased in round 3 these included new themes, suggesting instability. However, agreement increased for this statement. Both mean and median values showed the importance ranking lower but the range and SD both showed a move towards consensus.

**Table 11:** Agreement and importance values for statement 8

		Round 1	Round 2	Round 3
**Agreement**	Strongly agree	17%	30%	25%
	Agree	50%	50%	75%
	No opinion	25%	20%	0%
	Disagree	8%	0%	0%
	Strongly disagree	0%	0%	0%
**Importance**	Median	6	6.5	7
	Range	3–8	3–8	5–8
	Rank	8	7	8
	Mean	5.92	6.30	6.17
	Standard deviation	1.56	1.77	1.11
	Rank	8	6	6

### Kappa agreement (Table 12)

In general, K-values increased between rounds when rounds progressed, and when adjacent rounds were compared as opposed to round 1 Vs round 3. Statement 7, round 1 Vs round 2 showed the least agreement, K = 0.313, yet was consistently ranked the most important. This apparent anomaly is discussed below. Statement 4 had the second lowest SD by round 3 yet never achieved a K > 0.5. Statement 1, 3 and 7 got the 3 highest Kappa values, comparing round 2 and 3.

**Table 12:** Kappa values for within-subject agreement in importance rankings between rounds of each statement

	Agreement between Rounds 1 and 2	Agreement between Rounds 2 and 3	Agreement between first and last round (1 and 3)
Statement 1	0.642	0.836	0.432
Statement 2	0.697	0.516	0.596
Statement 3	0.600	0.750	0.533
Statement 4	0.498	0.490	0.470
Statement 5	0.696	0.669	0.627
Statement 6	0.627	0.641	0.548
Statement 7	0.313	0.711	0.375
Statement 8	0.515	0.690	0.519

## Discussion

The Delphi results show a change in participants' views towards consensus and stability as indicated by a trend towards:

• an increase in percentage agreements

• convergence of importance rankings

• increase in Kappa values

• a decrease in comments as rounds progressed

An increase was observed in percentage agreements for all statements over the 3 rounds with only 2 statements (Tables 6 and 8) showing some disagreement by round 3, compared to 7 statements in round 1. This demonstrates the evolution of consensus. Statement 4 (Table 7) had the highest disagreement percentage in round 1, yet full agreement by round 3, demonstrating that views could alter considerably.

Oscillatory movements at individual and group level were consistent with those described by Scheibe *et al.*[16]. A deviation in the level of agreement, "strongly agree" or "agree," between rounds was evident in most statements, and was not dependent on changes to statement phrasing (Table 7). The phenomenon of 2 participants failing to respond to round 2 had a bearing on the oscillatory movement, their lack of response gave a misleading bias towards agreement (Table 6). Individually both these participants disagreed to this statement, demonstrating disadvantage in the use of percentages. It is worth noting that non-responders can impact significantly on the sample size when interpreting percentages and this could lead to misleading oscillatory movements as suggested by Scheibe *et al.*[16].

The median and mean values for importance show the group aggregate rank, whereas ranges and SD show the spread, i.e. disagreement [18] of the group's responses around that result. Both range and SD decreased as rounds progressed, showing centralisation of views i.e. increased agreement or convergence. Comparison of importance rankings shows similarity in medians and means, equal or within one interval difference (Tables 4, 5, 7, 8, 9 and 10). Differences of 2 intervals were shown for statements 3 and 8 (Tables 6 and 11), which were 2 of the statements consistently ranked the least important. These discrepancies between means and medians could be explained by participants giving less attention to statements they consider least important.

While a decrease in range generally reflected a decrease in SD, there was no direct relationship between them. For example, between rounds 2 and 3, in statement 6 the SDs decreased from 1.65 and 1.56 but the ranges stayed constant, at 3–8. This highlights the different information each provides, SDs give an indication of the aggregate judgement where as ranges summarise the outliers views. Also between statements, equal ranges were represented by different SDs. For example statements 2 and 3 had a range of 1–8 in round 1, but standard deviations of 2.50 and 2.27, respectively. These findings expand on Greatorex & Dexter's conclusion [18] that each individual Delphi requires acceptable values of both mean and SD to represent consensus, by identifying that each statement must have individual values to determine convergence. However, this has implications for Delphi research as:-

• there may be increased potential for bias as researchers will need to make individual judgements on acceptable convergence levels for each statement,

• Alternatively, preconceived levels of convergence to determine consensus, as suggested by Williams & Webb [15], would be difficult to predict.

When interpreting the mean and median importance rankings, some between-test validity can be demonstrated. For example, when the mean and median were equal the lowest ranges and SDs were observed (Tables 4 and 7). Adding and subtracting the SD from the mean more accurately mirrored the range as the rounds progressed (table 4). As the SD represents the majority of subjects' variation around the mean, this shows there were fewer outliers as rounds progressed, again indicating lower disagreement [18], or increased convergence.

As expected, the greatest difference between K-values was between round 1 and 3 (Table 12), with K-values nearing 1, and greater agreement for round 2 versus 3. Observed points from K-values show no association with the ranges, SDs, medians, means or ranks. Possible explanations for this are the elimination of chance in Kappa and the comparison of *between *rounds in Kappa rather than values *within *a particular round. Also Kappa-crosstabs plot individuals' rank from one round against their rank in the next round, therefore K-values measure the value of agreement for *individuals between *two rounds, not agreement between *different *participants *within *a round. It was interesting to observe the apparent contradiction, between the results for Kappa and percentage agreement for statement 7. This may have been due to the small range of answers as compared with other statements, because Kappa is affected by range. This highlights the need to use a range of descriptive statistics in Delphi analysis.

Generally the number of comments decreased in each round. However, statement 8 (Table 11) did not show a decrease in comments but followed similar quantitative data patterns as other statements. One participant did comment on misunderstanding statement 8 in round 1, misunderstanding of the statement by participants possibly leading to the equivalent of a loss of a Delphi round for that statement until the meaning was clarified. This is validated by the rise in SD and range (Table 11), and rise in SD for statement 4, which was also misunderstood in round 1 (Table 7).

New themes were generated in round 3 for the 4 statements of least importance (Tables 6, 8, 9 and 11), suggesting instability, as views had not been exhausted. This was sometimes contradictory to the SDs and ranges which showed low scores, however they were still converging/lowering therefore showing instability. Although aggregating judgements will reduce the strength of the outliers views in quantitative data, with subjective views outliers can raise important issues not yet considered by others, and therefore new themes should not be ignored and rounds continued until views are exhausted.

Statements 1 and 4 which received fewer comments in round 3 had the lowest SDs. Because quantitative analysis aggregates participants' judgements, outlying individual judgements are not represented. However, the reduction in comments does support the lowering of range intervals and SD, substantiating the evolution of consensus and validating quantitative data with qualitative data.

The results presented here demonstrate two points that can be used to improve Delphi studies in future. Firstly, the results have demonstrated that the mean and SD, when combined with the range and medians, can be used to show whether convergence has occurred, by a movement towards central tendency. This is in agreement with the findings of Greatorex and Dexter [18]. The amount of convergence and therefore the strength of agreement is indicated by a comparison of SD (strength of aggregate judgement) and range (larger ranges being indicative of outliers views). Secondly, high or increasing Kappa values demonstrate stability of individuals' views within the group and the level of agreement between rounds. Furthermore, reductions in the number of subjective comments reinforce the quantitative observations of convergence. It is rare to see this in Delphi studies and it is proposed that in future Delphi studies, this analytical combination is used.

There are limitations to this study. This study used a small sample size therefore the results should be treated with some caution, and followed up by a larger study. However this offered the greatest opportunity for analysing all qualitative data, because an increased sample size would have led to saturation of data, and it is not uncommon for Delphi studies to use this type of sample size.

## Conclusion

Using standard descriptive statistics and Kappa calculations in conjunction with thematic analysis and the number of comments generated, it was possible to demonstrate movement towards consensus and stability in this Delphi study. Following the original use of Delphi in social science, Delphi is suggested to be an effective way to gain and measure group consensus in healthcare [1]. There is potential here to add clarification to the use of a very subjective methodology. It is suggested that a combination of the simple descriptive statistics as presented here be used to reduce subjectivity and ensure maximum validity of results in Delphi methodology for improved evidence of consensual decision-making. The trends observed in this exploratory study suggest that a larger study is warranted, following the same approach.

## Competing interests

The author(s) declare that they have no competing interests.

## Authors' contributions

JLF and EAH designed the study. JLF collected the data. All authors participated in the analysis and interpretation of the data, and the drafting, progress and revision of the manuscript.

## Pre-publication history

The pre-publication history for this paper can be accessed here:


